# Retinitis Pigmentosa with *EYS* Mutations Is the Most Prevalent Inherited Retinal Dystrophy in Japanese Populations

**DOI:** 10.1155/2015/819760

**Published:** 2015-06-16

**Authors:** Yuuki Arai, Akiko Maeda, Yasuhiko Hirami, Chie Ishigami, Shinji Kosugi, Michiko Mandai, Yasuo Kurimoto, Masayo Takahashi

**Affiliations:** ^1^Laboratory for Retinal Regeneration, Center for Developmental Biology, RIKEN, Kobe 650-0047, Japan; ^2^Department of Ophthalmology, Case Western Reserve University, Cleveland, OH 44124, USA; ^3^Department of Pharmacology, Case Western Reserve University, Cleveland, OH 44124, USA; ^4^Institute of Biomedical Research Innovation Hospital, Kobe 650-0047, Japan; ^5^Department of Medical Ethics/Medical Genetics, Kyoto University School of Public Health, Kyoto 606-8501, Japan; ^6^Kobe City Medical Center General Hospital, Kobe 650-0047, Japan

## Abstract

The aim of this study was to gain information about disease prevalence and to identify the responsible genes for inherited retinal dystrophies (IRD) in Japanese populations. Clinical and molecular evaluations were performed on 349 patients with IRD. For segregation analyses, 63 of their family members were employed. Bioinformatics data from 1,208 Japanese individuals were used as controls. Molecular diagnosis was obtained by direct sequencing in a stepwise fashion utilizing one or two panels of 15 and 27 genes for retinitis pigmentosa patients. If a specific clinical diagnosis was suspected, direct sequencing of disease-specific genes, that is, *ABCA4* for Stargardt disease, was conducted. Limited availability of intrafamily information and decreasing family size hampered identifying inherited patterns. Differential disease profiles with lower prevalence of Stargardt disease from European and North American populations were obtained. We found 205 sequence variants in 159 of 349 probands with an identification rate of 45.6%. This study found 43 novel sequence variants. In silico analysis suggests that 20 of 25 novel missense variants are pathogenic. *EYS* mutations had the highest prevalence at 23.5%. c.4957_4958insA and c.8868C>A were the two major *EYS* mutations identified in this cohort. *EYS* mutations are the most prevalent among Japanese patients with IRD.

## 1. Introduction

Retinitis pigmentosa (RP) is the most common form of inherited retinal dystrophies (IRD) and is clinically and genetically heterogeneous. At least 50 genes have been identified for nonsyndromic RP [1] (RetNet; http://sph.uth.tmc.edu/RetNet/ provided in the public domain by the University of Texas Houston Health Science Center, Houston, TX). They include genes required for phototransduction, visual cycle, cilial transportation in photoreceptors, and maintenance of photoreceptor structure [2, 3]. RP patients commonly display a disease progression profile beginning with rod photoreceptor degeneration followed by cone photoreceptor death; hence patients with RP present clinically with night blindness and progressive restriction of the visual field followed by impairment of central and color vision. The prevalence of RP has been reported at 1 in 3,000-4,000 individulas worldwide [2] and a similar rate is expected in Japanese populations. Autosomal dominant (adRP), autosomal recessive (arRP), and X-linked (xlRP) patterns of inheritance are common in RP. Within Japanese populations identifiable inheritance patterns are recognized in nearly 50% of all RP cases with 35%, 10%, and 5% in arRP, adRP, and xlRP, respectively. The remaining 50% of cases are considered simplex or sporadic. In the clinic, gaining information about a mode of inheritance is limited due to recent social trends including decreasing family size and increasing social isolation. Therefore, establishment of molecular diagnoses for patients with unknown disease inheritance is critical. We reported in 2008 the first comprehensive molecular diagnosis for RP in Japanese patients with known and unknown inheritance and identified 26 mutations in 28 of 209 probands (203 of RP, 2 of areolar atrophy, 3 of cone dystrophy, and 1 of Stargardt disease) [4]. To accomplish this study, we employed a method which combined an efficient denaturing high performance liquid chromatography (dHPLC) based assay with 108 exons of 30 RP-causing genes and confirmative direct DNA sequencing.

In the present study, we further increased the screening number of the genes and exons, and another cohort of 349 Japanese patients was examined to gain additional information about disease prevalence and to identify the responsible genes for IRD in Japanese populations. For this purpose, direct sequencing of stepwise analyses utilizing one or two panels of 15 and 27 genes was conducted for RP cases. Disease-specific genes were also analyzed on patients with other IRD.

## 2. Materials and Methods

### 2.1. Patients and Families

We performed mutation analysis in a cohort of Japanese patients with IRD, who visited the RP/Genetic Counseling Clinic in the Institute of Biomedical Research and Innovation Hospital, Kobe, Japan, from October 2008 to May 2014. A total of 412 individuals, 349 probands, and their 26 affected and 37 unaffected family members were involved in this study. The Human Genetic Variation Database from 1,208 Japanese individuals provided by Japanese genetic variation consortium, Kyoto University, Kyoto, Japan, was used as controls (http://www.genome.med.kyoto-u.ac.jp/SnpDB/) [5]. None of the patients in this cohort were enrolled in our previous study [4]. Informed consent was obtained from all patients and family members after the genetic screening procedures had been fully explained. Research protocols were approved by the institutional review boards of the RIKEN and the local research ethics committees, and the study was conducted in accordance with the principals of the Declaration of Helsinki.

### 2.2. Mutation Analysis

**Figure 1:**
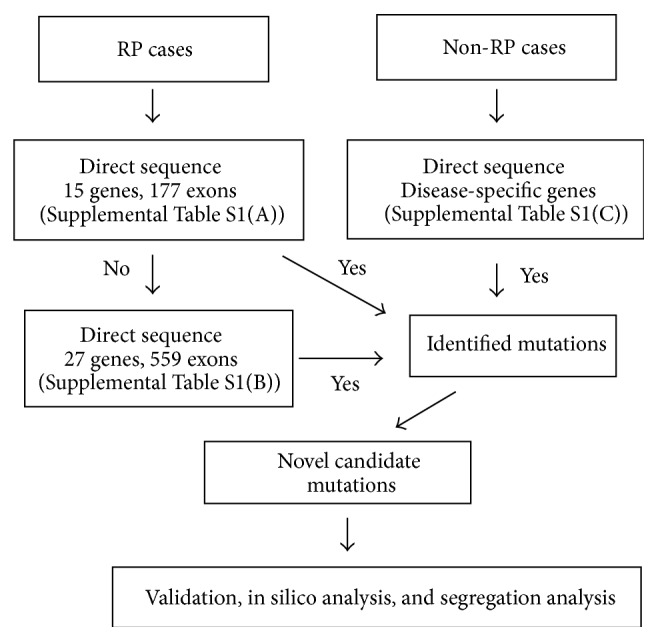
A stepwise screening for patients with IRD. Molecular diagnosis was performed with a stepwise screening methodology. Patients with RP were initially screened with 15 genes, and additional 27 genes were sequenced when the initial screening failed to detect mutations. Disease-specific genes were sequenced for patients with other IRD.

Genomic DNA was extracted from peripheral lymphocytes using standard procedures. Mutation screening was performed by a stepwise direct sequencing utilizing one or two panels of genes listed in Supplemental Tables S1(A) and S1(B) in Supplementary Material available online at http://dx.doi.org/10.1155/2015/819760 for patients with RP (Figure 1). Only patients whose sequence variants were not detectable underwent additional screening with the 2nd panel. If a specific clinical diagnosis was suspected, direct sequencing of disease-specific genes was conducted by analyzing the following genes for mutations (Supplemental Table S1(C)):* ABCA4* and* RDS/PRPH2* for Stargardt disease,* CHM* for choroideremia,* CYP4V2* for Bietti crystalline dystrophy,* SAG* for Oguchi disease,* VMD2* for Best disease,* USH2A* for Usher syndrome,* RS1* for retinoschisis,* RDS/PRPH2* for central areolar choroidal dystrophy, and* RDH5* and* RLBP1* for fundus albipunctatus. In silico analysis was performed to evaluate the potential deleterious effects of novel missense mutations utilizing the following four computational prediction algorithms: PolyPhen2 (http://genetics.bwh.harvard.edu/pph2/index.shtml), SIFT (http://sift.jcvi.org/), PMut (http://mmb2.pcb.ub.es:8080/PMut/PMut.jsp), and SNAP (https://rostlab.org/services/snap/). Variants were determined to carry potential deleterious effects when 50% and higher rates of the programs predict their pathogenicity. Segregation analysis was also applied to families with newly identified mutations.

### 2.3. Clinical Diagnosis

Full medical and family histories were taken, pedigrees were drawn, and ophthalmologic examinations were performed for each patient. Clinical evaluation included best correct visual acuity (BCVA) according to projected Snellen charts, slit-lamp biomicroscopy, and dilated indirect ophthalmoscopy. Retinal imaging using a Topcon TRC-NW7SF retinal camera (Topcon Corporation, Tokyo, Japan), optical coherence tomography, and retinal autofluorescence imaging using a SPECTRALIS_Spectral domain optical coherence tomography (OCT) scanner (Heidelberg Engineering, Heidelberg, Germany) were conducted. Full-field electroretinogram (ERG) was also performed. The ERG protocol complied with the standards published by the International Society for Clinical Electrophysiology of Vision. Visual fields were examined with Goldmann perimetry.

## 3. Results and Discussion

### 3.1. A High Rate of Clinical Diagnosis as RP in This IRD Cohort

**Figure 2:**
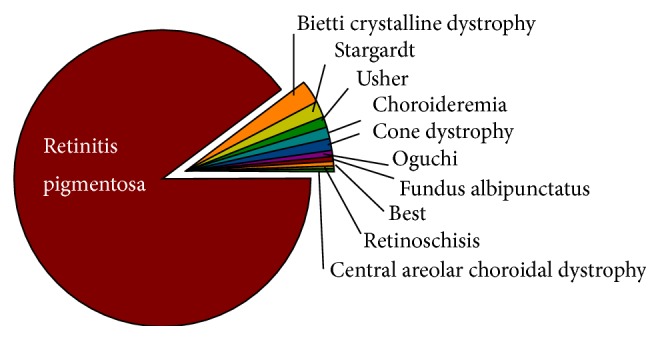
Retinal dystrophies included in this study. Clinical diagnosis of each retinal disease is shown. Nonsyndromic RP was found in 313 of 349 cases at the rate of 89.6%. We revised the clinical diagnosis in one case after genotyping: the patient diagnosed with cone dystrophy was revised to autosomal recessive enhanced S-cone syndrome (ESC) due to compound heterozygous* NR2E3* mutations: c.419A>G and c.488T>C.

The summary of clinical diagnoses in our cohort is shown in Figure 2, and 313 of 349 (89.6%) patients were diagnosed as nonsyndromic RP. The fraction of Stargardt disease patients in our cohort was 1.2%. The estimated prevalence of Stargardt disease is 1 in 8,000–10,000 individulas in North America [6] and that of RP is 1 in 3,000-4,000 individulas [2]. The estimated prevalence of RP in Japan is also 1 of 3,000-4,000 individulas; however, our data indicates a higher rate of RP and a lower prevalence of Stargardt disease in Japanese IRD populations. Additionally choroideremia, in which we identified 5 genetically unrelated patients in this study, has a reported prevalence of 1 in 50,000–100,000 individulas in North America (http://ghr.nlm.nih.gov/condition/choroideremia). Usher syndrome was found in 5 unrelated patients and was the only syndromic disease identified. Bietti crystalline dystrophy (BCD) is known to be more common in people with East Asian ancestry [7], and 11 unrelated BCD patients were identified in this cohort. Although the high RP and low Stargardt disease prevalence might be associated with patient referral bias to our clinic, the RP/Genetic Counseling Clinic, the current study clearly demonstrates differential disease profiles between racial backgrounds. We revised the clinical diagnosis in one case after genotyping: the patient diagnosed with cone dystrophy was revised to autosomal recessive enhanced S-cone syndrome (ESC) due to compound heterozygous* NR2E3* mutations: c.419A>G and c.488T>C.

### 3.2. High Prevalence of* EYS* Mutations in Japanese Cases

Genetic testing is recommended for patients with IRD, because the results gained can make a positive impact on both patients and their families [8]. Identification of the causative genes leads to improved accuracy of diagnoses, providing patients prognostic information and better genetic counseling, and can facilitate further research in the development of mechanism-specific care. In this study we analyzed genetic mutations using the stepwise direct sequencing of the majority of coding sequence in genes which are known to cause RP and other IRD (Figure 1 and Supplemental Tables S1(A) and S1(B)). Only patients whose sequence variants were not detectable underwent additional screening with the 2nd panel. If a specific clinical diagnosis was suspected, direct sequencing of disease-specific genes was conducted by analyzing the following genes for mutations (Supplemental Table S1(C)).

Inherited patterns of IRD in this cohort are summarized in Table 1. A total of 205 changes in 26 genes were detected as summarized in Table 2. These 205 sequence variants were found in 159 of 349 probands (45.3%). These patients carried between 1 and 4 sequence variants. Surprisingly, 134 of 303 or 44.2% of autosomal recessive or simplex cases were identified to carry sequence variants, and this rate was higher than that of X-linked retinal dystrophies with the detection rate of 36.3%. The identification rate of xlRP was reported as 35% in cohorts of North American populations [9]. This study was designed and conducted as a continuation of our previous study which utilized a dHPLC based assay [4], and we also expanded the members of genes and exons examined. These changes successfully contributed to a better identification rate as compared to our previous study from 13.4% to 45.3%.

Sequence variants in* EYS* were the most frequent in our cohort and were detected in 82 of 349 probands (23.5%). The second most frequently mutated gene in our cohort was* RDS/PRPH2* and accounted for 4.6% of cases. Mutations in* EYS* were found in 32.8% [10] and 16% [11] of Japanese arRP patients. Patients with compound heterozygous* EYS *mutations were summarized in Supplemental Table S2.* EYS* was identified in Spanish patients with arRP in 2008 [12], and indeed* EYS* was not tested in our 2008 study. The detection rate of this current study would drop to 20.6% if* EYS* was excluded from our current analysis. As compared to recently published Japanese cohort studies [13, 14], this study displayed a lower detection rate of* USH2A*. This is probably due to our two-panel screening methodology. Since* USH2A* is included in the 2nd panel, this gene was not examined if the 1st panel detected sequence variation(s). Therefore, data interpretation needed to be performed cautiously and special consideration had to be made to the strengths and limitations of each method.

Another significant finding is that this study found a mutation c.469G>A (p.G157R) in* GUCA1B* in 4 patients with RP. The mutation in* GUCA1B* was found only in 3 Japanese adRP families [15] and no mutations in* GUCA1B* have ever been found in 400 British patients with adRP [16]. A more recent study failed to detect* GUCA1B* mutations in patients with cone dystrophy and cone-rod dystrophy, and thus the authors concluded that* GUCA1B* is a minor cause for IRD in Europeans and North Americans [17]. Given the result of 4 unrelated RP patients carrying the previously reported c.469G>A mutation in this cohort, this mutation could be frequent at least for the Japanese IRD populations.

### 3.3. Novel Sequence Variants Identified in this Study

**Figure 3:**
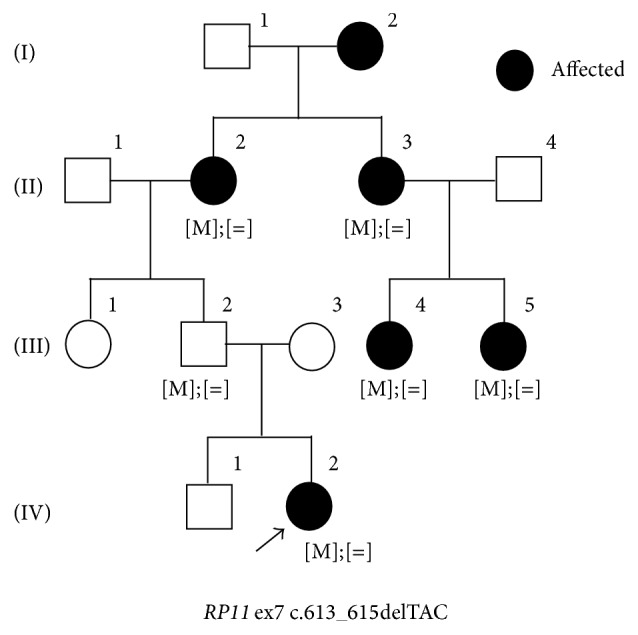
A pedigree of adRP with the novel* RP11*  mutation. A family carrying a novel c.613_615delTAC mutation in* RP11* is presented. All of 6 family members who underwent molecular diagnosis carried the heterozygous c.613_615delTAC mutation as indicated with [M];[ = ]. Affected individuals are indicated as filled symbols, and an arrow indicates the proband in this family.

**Figure 4:**
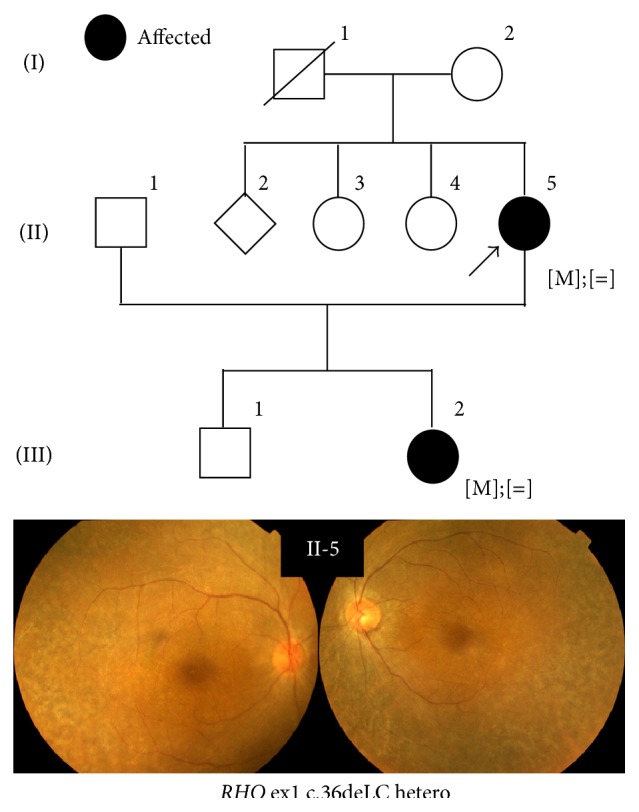
A pedigree of probable adRP with a novel* RHO* mutation. A family with a novel* RHO* mutation c.36delC is shown. II-5 in this family carried the heterozygote mutation, and her clinical phenotype was relatively mild with late onset at her age of 62. III-2 showed only marginal clinical signs of RP when she underwent clinical evaluations at the age of 44. A possible carrier of I-1 died before the age when the III-2 presented RP symptoms. Affected individuals are indicated as filled symbols, and an arrow indicates the proband in this family. Bottom images are fundus pictures of II-5.

**Figure 5:**
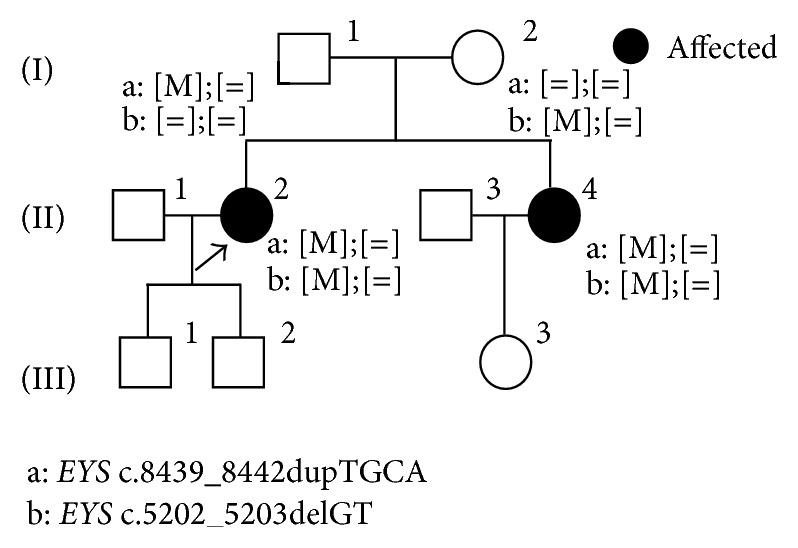
A pedigree of arRP with novel* EYS *mutations. A family carrying two novel* EYS *mutations, c.8439_8442dupTGCA (a) and c.5202_5203delGT (b), is presented. Affected II-2 and II-4 carried compound heterozygous mutations. I-1 carried a heterozygous c.8439_8442dupTGCA mutation and I-2 did another c.5202_5203delGT heterozygous mutation. Affected individuals are indicated as filled symbols, and an arrow indicates the proband in this family.

This study revealed 43 novel sequence variants including 32* EYS* mutations (Tables 3–5). A total of 11 novel sequence variants were identified in the following genes:* ABCA4*,* CRX*,* PROM1*,* RDS/PRPH2*,* RHO*, and* RP11* (Table 3). The same novel alteration, c.613_615delTAC in* RP11*, was found in two unrelated families. As shown in Figure 3, segregation analyses revealed that this novel mutation contributed to adRP in this family with III-2 as a nonpenetrant. The same novel c.1738A>C alteration was detected in* PROM1* in three unrelated families (this variant is described more in the section of “Association of* EYS*,* CRB1*, and* PROM1 *in retinal dystrophy”). This cohort also included a family with a novel* RHO* sequence variant, c.36delC (Figure 4). II-5 in this family carried the heterozygous mutation, and her clinical phenotype was relatively mild with late onset at the age of 62. III-2 showed only marginal clinical signs of RP when she had underwent clinical evaluations at the age of 44. A possible carrier of this mutation, I-1, died before the age when III-2 presented RP symptoms. This truncating variant is located in exon 1 and it is likely to contribute to adRP. A total of 13 novel truncating* EYS* sequence variants were found in 21 RP patients in 13 families (Table 4) and 19 missense changes in* EYS* were also recognized (Table 5). Two novel* EYS *mutations, c.8439_8442dupTGCA and c.5202_5203delGT, were found in a single family (Figure 5). Affected family members II-2 and II-4 carried the compound heterozygous mutations. In silico analysis suggests that 20 of 25 novel missense mutations identified here harbor potential deleterious effects (Table 5), and further segregation analyses are essential to conclude their pathogenesis.

### 3.4. c.4957_4958insA and c.8868C>A Are the Two Major* EYS* Mutations in Japanese Patients with RP

*EYS* alterations were detected in 126 alleles in 82 probands with the highest prevalence in this Japanese cohort of IRD (Table 2). All* EYS* sequence variants were found in patients with nonsyndromic RP. We found 4 major* EYS* variants which were detected in unrelated families. Mutations of c.4957_4958insA in exon 26 and c.8868C>A in exon 44 were the two highest prevalent* EYS* mutations observed (Table 6). The frequencies of c.4957_4958insA and c.8868C>A variants were 26.8% (44 alleles in 82 probands with* EYS* mutations) and 13.4% (22 of 82), respectively. No c.4957_4958insA mutation has been detected in 1,208 Japanese individuals without eye symptoms. These data suggest that screening and developing treatment options for these two mutations, c.4957_4958insA and c.8868C>A, can greatly improve RP care in Japan.

*EYS *was previously known as the* RP25* gene located at a 16 cM region on chromosome 6p12.1–q15 [18], and linkage to the same locus was reported in multiple families from various ancestral origins including Spanish [18], Pakistani [19], and Chinese [20]. The* RP25 *was identified as* EYS* in Spanish patients with arRP in 2008 [12]. Additional studies found that mutations in* EYS* are the major cause of arRP in Spanish [21], Chinese and British [22], and Israeli and Palestinian populations [23]. In contrast, prevalence of* EYS* mutations was lower in other ethnic groups, and they account for approximately 5% of arRP patients of Western European ancestry [24]. These two major mutations, c.4957_4958insA and c.8868C>A, have been only found in Japanese and Korean populations [11].

#### 3.4.1. Association of* EYS*,* CRB1*, and* PROM1* in Retinal Dystrophies

*EYS* was identified in* Drosophila* mutants with a compromised optomotor response [25] and characterized as a molecule which plays an important role for producing an interrhabdomeral space in the* Drosophila* retina [26]. Recent study with* Drosophila* models revealed that* eys* forms a genetic network with* chaoptin*,* prominin,* and* crumbs* for controlling the apical compartment of their photoreceptor cells [27]. Interestingly, the same study reported that deficiency of* eys*,* prominin, *or* crumbs* in flies displayed light-induced photoreceptor degeneration. Furthermore, light-induced photoreceptor death in these three mutants was rescued by culturing under vitamin A deficient conditions. Mutations of human homologs of these three genes,* EYS *[12],* PROM1 *[28], and* CRB1 *[29], can cause RP. All of these observations led us to speculate that RP caused by these genes could share disease pathogenesis. Simultaneously such observations also suggest possible digenic and polygenic diseases due to combination(s) of their impairments as observed in* ROM1* and* RDS/PRPH2* mutations [30].

Therefore, we examined the association of these three genes,* EYS*,* PROM1*, and* CRB1*, in 12 RP patients with a heterozygous* EYS* mutation and two family members (Table 7). This additional screening found a novel heterozygous probable pathogenic* PROM1* mutation c.1738A>C in two patients. Notably,* PROM1* mutations have been reported to cause autosomal dominant cone dystrophy [31], and, indeed, the heterozygous mutation was found in our autosomal dominant cone dystrophy (Table 3).* PROM1* mutations can cause arRP [32]. Furthermore, these two patients also carried a* CRB1* sequence variant c.2306G>A (rs62636287 in NCBI database) whereas 1,208 control subjects did not. c.2306G>A was also found in a healthy family member without carrying a heterozygous* EYS* mutation. In the neighboring area of c.2306G>A, c.2302C>T an unreported variant, c.2303G>A, and c.2303C>T reported nonpathogenic variants were identified in 2, 6, and 3 control individuals, respectively. Moreover, 5 of 12 patients carried c.2306G>A in addition to a heterozygous* EYS* mutation. These results suggest the possibility of digenic and polygenic diseases due to combination(s) of* EYS*,* PROM1*, and* CRB1 *mutations. It is worth noting that copy number variants (CNVs) in* EYS *were reported in patients with IRD [33], and, therefore, molecular diagnosis of* EYS *should include analysis for CNVs. Our study observations and the relatively high prevalence of known recessive RP mutations in the general population [34] could produce additional complexity and difficulty in genetic counseling for arRP cases.

## 4. Conclusion

Molecular diagnosis of patients from different ethnic backgrounds greatly contributes to our understanding of the global spectrum of human disease-causing mutations and helps to develop therapies. Considering the possible number of therapeutic targets and relatively small number of patient populations with IRD, it is essential to define which genes can be the candidates for developing new therapies and which genes could more efficiently bring benefits to more patients worldwide. This study revealed different patterns of disease and genetic prevalence in Japanese populations as compared to European and North American populations. Most significantly Japanese populations have a higher prevalence of* EYS* mutations (23.5%) and less rhodopsin mutations (2.0%) compared to European and North American populations. In our Japanese cohort, we did not find the most common European origin P23H of* RHO* mutation in adRP [35]. Additionally, we found a higher rate of RP and a lower prevalence of Stargardt disease in this Japanese cohort. Drawbacks of this study exist including patient referral bias and the heterogeneous disease presentation due to the wide range of clinical phenotypes seen in Stargardt disease [36, 37] and other IRD; nevertheless the data presented here demonstrates clear differences in causative genetic mutations between racial backgrounds.

In order to further increase the rate of identification of pathogenic mutations in IRD, other methods could be employed, such as entire exome sequencing using next generation sequencing (NGS), which utilizes a greater genetic panel for screening. Currently, both targeted and whole exome or genome analyses combined with NGS are becoming prevalent and several analyzers on the market reduce both time of analysis and cost of sequencing [38–40]. Because such NGS can also be easily shifted to and combined with RNA sequence analysis, understanding of pathogenesis due to specific mutations can be vastly improved. Furthermore, this study and other studies suggest that* EYS* screening for all RP,* RPGR*, and* RP2* for xlRP and male simplex RP [9] should be included as first tier assessment for Japanese patients with RP.

In summary, we performed the largest comprehensive mutational analysis on IRD in a Japanese population cohort. We identified 205 sequence variants in 349 probands and found a high prevalence of mutations in* EYS*. Furthermore, this is the first study examining a spectrum of IRD in Japanese cases and it provided additional evidence of differential disease prevalence among races.

## Supplementary Material

Supplemental Table 1: Tested genes in this study are summarized.
Supplemental Table 2: Patients with compound heterozygous EYS mutations.

## Figures and Tables

**Table 1:** Inherited patterns and diagnostic rates.

Inheritance	Tested (*n*)	Detected (*n*)	Detected (%)
Autosomal dominant	35	21	60.0
Autosomal recessive/simplex	303	134	44.2
X-linked	11	4	36.3

	349	159	45.6

**Table 2:** Prevalence of mutations among 349 probands in this study.

Gene	Location	Probands (*n*)	Prevalence (%)	Disease category
*EYS *	6q12	82	23.5	arRP
*RDS/PRPH2 *	6p21.1	16	4.6	adRP, arRP, adMD^*^
*RHO *	3q22.1	7	2.0	adRP, arRP
*CYP4V2 *	4q35.2	7	2.0	Bietti crystalline dystrophy
*CRB1 *	1q31.3	5	1.4	arRP, LCA
*RP11 *	19q13.42	4	1.2	adRP
*GUCA1B *	6p21.1	4	1.2	adRP
*PROM1 *	11q12.3	4	1.2	adRP
*RPGR *	Xp11.4	4	1.2	xlRP
*ABCA4 *	1p22.1	3	0.9	STGD^†^
*ROM1 *	11q12.3	3	0.9	adRP, arRP
*CRX *	19q13.32	2	0.6	adRP, CORD^‡^
*CHM *	Xq21.2	2	0.6	Choroideremia
*GUCY2D *	17p13.1	2	0.6	arRP
*RP2 *	Xp11.23	2	0.6	xlRP
*RP9 *	7p14.3	2	0.6	adRP
*TOPORS *	9p21.1	2	0.6	adRP
*USH2A *	1q41	2	0.6	arRP
*CNGB3 *	8q21.3	1	0.3	Cone dystrophy
*IMPDH1/RP10 *	7q32.1	1	0.3	adRP
*MAK *	6p24.2	1	0.3	arRP
*NR2E3 *	15q23	1	0.3	ESC^§^
*RDH5 *	12q13.2	1	0.3	FA^#^
*RP1 *	8q12.1	1	0.3	adRP, arRP
*RLBP1 *	15q26.1	1	0.3	arRP
*SAG *	2q37.1	1	0.3	Oguchi disease

205 gene alterations were found in 349 probands.

^*^MD: macular dystrophy.

^†^STGD: Stargardt disease.

^‡^CORD: cone-rod dystrophy.

^§^ESC: enhanced S-cone syndrome.

^#^FA: fundus albipunctatus.

**Table 3:** Clinical characterization of patients with novel mutations in this study (except* EYS*).

Patient	Gene	DNA variant	Protein variant	Diagnosis	Age of onset	Age at exam	VA_OD	VA_OU	RE_OD (diopter)	RE_OU (diopter)	Fundus	Visual field
1	***ABCA4***	c.2593_2594insT	p.Y865L fs^*^20	STGD	10	36	0.03	0.01	nc	nc	Macular degeneration	Peripheral islands

2	***CRX***	c.118C>T	p.R40W	CORD	65	70	0.15	0.2	+1.0	+0.75	Perimacular RPE atrophy	Central scotoma

3	***PROM1***	c.1738A>C	p.N580H	CD	37	37	1.2	0.3	−3.5	−3.0	Macular degeneration	Central scotoma

4	***RDS/PRPH2***	c.346G>T	p.A116S	RP	10	45	1.2	1.2	nc	nc	Perimacular AF	30°
5		c.44A>G	p.K15R	RP	na	na	LP	0.2	nc	−2.5	WSA	10°
6		c.460A>C	p.K154Q	RP	15	na	0.4	0.3	−8.5	−7.0	WSA, BS, NV	na

7	***RHO***	c.302G>A	p.G101E	RP	10	52	1.2	1.2	+1.0	+1.75	WSA, BS, NV	na
8		c.36delC	p.P12S fs^*^35	RP	62	74	0.8	0.9	nc	nc	WSA	30°

9	***RP11***	c.523delIC	p.Q175R fs^*^23	RP	16	48	0.6	0.02	+0.25	nc	WSA, BS, NV	10°
10		c.1140_114insTC	p.G381S fs^*^33	RP	18	58	0.5	0.5	+0.5	+1.5	WSA, BS, NV	10°
11		c.613_615delTAC	p.Y205^*^	RP	10	66	HM	LP	nc	nc	WSA, BS, NV	10°

*RP11*, c.613_615delTAC mutation was found in 6 patients of 2 families.

*PROM1*, c.1738A>C mutation was found in other 2 RP patients with heterologous *EYS * and *CRB1 * alterations (see Table 7).

Patient 6 had antirecoverin Ab.

^*^Truncating and nonsense variants.

VA: visual acuity.

RE: refractive error.

nc: not correctable.

na: not available.

LP: light perception.

WSA: widespread RPE atrophy.

BS: bone-spickle.

NV: narrow vasculature.

HM: hand motion.

**Table 4:** Novel truncating and nonsense *EYS* mutations found in this study.

Mutation	DNA variant	Protein variant	Diagnosis
1	c.179delT	p.L60W fs^*^3	RP
2	IVS27-3_4insT	—	RP
3	IVS38-1G>T	—	RP
4	c.2380C>T	p.R794^*^	RP
5	c.4557delA	p.A1520P fs^*^30	RP
6	c.5202_5203delGT	p.F1735Q fs^*^6	RP
7	c.6869_6896delCCATATTCCTGCAAATGTTCAAATTGATAAGAAAG	p.P2290Q fs^*^12	RP
8	c.6897_6902dupAGGTCC	p.G2300_P2301dup	RP
9	c.6976C>T	p.R2326^*^	RP
10	c.7836_7837delTC	p.P2613L fs^*^18	RP
11	c.8196_8200delCTTTC	p.F2733C fs^*^33	RP
12	c.8439_8442dupTGCA	p.E2815C fs^*^19	RP
13	c.8921C>A	p.S2974^*^	RP

^*^Truncating and nonsense variants.

**Table 5:** In silico analysis for novel missense mutations.

Gene	Protein variant	DNA variant	Prediction for damage
*CRX *	p.R40W	c.118C>T	**4 of 4**
*EYS *	p.K4E	c.10A>G	**2 of 4**
*EYS *	p.R26Q	c.77G>A	**2 of 4**
*EYS *	p.M12T	c.35T>C	**2 of 4**
*EYS *	p.E47D	c.141A>T	**3 of 4**
*EYS *	p.Q76H	c.228G>C	**2 of 4**
*EYS *	p.C211Y	c.632G>A	**4 of 4**
*EYS *	p.I256M	c.768A>G	**2 of 4**
*EYS *	p.G484R	c.1450G>A	**3 of 4**
*EYS *	p.N1205T	c.3614A>C	**2 of 4**
*EYS *	p.K1633E	c.4897A>G	**2 of 4**
*EYS *	p.L1655M	c.4963 T>A	**2 of 4**
*EYS *	p.L1802F	c.5404C>T	**2 of 4**
*EYS *	p.G2186E	c.6557G>A	**2 of 4**
*EYS *	p.I2188T	c.6563T>C	1 of 4
*EYS *	p.R2604C	c.7810C>T	**2 of 4**
*EYS *	p.T2683I	c.8048C>T	0 of 4
*EYS *	p.D2767H	c.8299G>C	**3 of 4**
*EYS *	p.L2784R	c.8351T>G	**2 of 4**
*EYS *	p.I3091T	c.9272T>C	**2 of 4**
*PROM1 *	p.N580H	c.1738A>C	**3 of 4**
*RDS *	p.K15R	c.44A>G	1 of 3
*RDS *	p.A116S	c.346G>T	0 of 4
*RDS *	p.K154Q	c.460A>C	0 of 4
*RHO *	p.G101E	c.302G>A	**4 of 4**

Bold indicates in silico analysis indicates pathogenic higher than 50% rates.

**Table 6:** Frequency of major *EYS* variants in 82 probands with *EYS* mutations.

	Exon	DNA variant	Protein variant	Allele	Frequency
*EYS *	ex26	c.4957_4958insA	p.S1653K fs^*^2	44	26.8
*EYS *	ex32	c.6557G>A	p.G2186E	7	4.3
*EYS *	ex32	c.6563T>C	p.I2188T	8	4.9
*EYS *	ex44	c.8868C>A	p.Y2956^*^	22	13.4

^*^Truncating and nonsense variants.

**Table 7:** Association of *EYS*, *PROM1,* and *CRB1* in retinal dystrophies.

*EYS *	PROM1	*CRB1 *	Diagnosis
c.768A>G (p.I256M)	c.1738A>C (p.N580H)	c.2306G>A (p.R769H)	adRP
c.4957_4958insA	c.1738A>C (p.N580H)	c.2306G>A (p.R769H)	adRP
c.1450G>A (p.G484R)	n.d.	c.2306G>A (p.R769H)	adRP
c.8868C>A (p.Y2956^*^)	n.d.	c.2306G>A (p.R769H)	adRP
c.4963T>A (p.L1655M)	n.d.	c.2306G>A (p.R769H)	arRP

n.d.	n.d.	c.2306G>A (p.R769H)	(Normal)

12 patients with RP were examined.

^*^Truncating and nonsense variants.

adRP indicates likely autosomal dominant retinitis pigmentosa.

n.d. indicates not detected.

## Abbreviations

ABCA4: ATP-binging cassette transporter, family 4
CHM: Choroideremia gene
CYP4V2: Cytochrome P450, family 4, subfamily V, polypeptide 2
EYS: Eyes shut homolog
IRD: Inherited retinal dystrophy
PRPH2: Peripherin 2
RDH5: Retinol dehydrogenase 5
RDS: Retinal degeneration slow
RHO: Rhodopsin
RLBP1: Retinol binding protein 1
RP: Retinitis pigmentosa
RS1: Retinoschisis 1
SAG: S-antigen or arrestin
USH2A: Usher syndrome 2A
VMD2: Vitelliform macular dystrophy 2.
