# Supplementary material for: Retinitis Pigmentosa with EYS Mutations Is the Most Prevalent Inherited Retinal Dystrophy in Japanese Populations
Source: J Ophthalmol. 2015 Jun 16;2015:819760. doi: 10.1155/2015/819760 (PMC4487330; doi:10.1155/2015/819760)
Supplement: Supplementary file 1 — Supplemental Table 1: Tested genes in this study are summarized. Supplemental Table 2: Patients with compound heterozygous EYS mutations. [file 819760.f1.pdf]

Supplemental Table S2. Patients with compound heterozygous *EYS* mutations

patient	location	DNA variant	Protein variant	SNP ID	MAF
1	ex32	c. 6563T>C	p. I2188T		0
	ex44	c. 8868C>A	p. Y2956*		0
2	ex26	c. 4957_4958insA	p. S1653Kfs*2		0
	ex32	c. 6557G>A	p. G2186E	rs74419361	0.0023
3	ex26	c. 4957_4958insA	p. S1653Kfs*2		0
	ex32	c. 6563T>C	p. I2188T		0
4	ex32	c. 6557G>A	p. G2186E	rs74419361	0.0023
	ex16	c. 2522_2523insA	p. Y841fs*		0
5	ex15	c. 2380C>T	p. R794*		0
	ex26	c. 4957_4958insA	p. S1653Kfs*2		0
6	ex26	c. 4957_4958insA	p. S1653Kfs*2		0
	ex44	c. 8868C>A	p. Y2956*		0
7	ex26	c. 4957_4958insA	p. S1653Kfs*2		0
	IVS38	c. -1G>T	p. -		0
8	ex26	c. 4957_4958insA	p. S1653Kfs*2		0
	ex44	c. 8868C>A	p. Y2956*		0
9	ex26	c. 4957_4958insA	p. S1653Kfs*2		0
	ex44	c. 8868C>A	p. Y2956*		0
10	ex32	c. 6557G>A	p. G2186E	rs74419361	0.0023
	ex44	c. 8868C>A	p. Y2956*		0
11	ex32	c. 6563T>C	p. I2188T		0
	ex44	c. 8868C>A	p. Y2956*		0
12	ex26	c. 4957_4958insA	p. S1653Kfs*2		0
	ex44	c. 8351T>G	P. L2784R		0
13	ex26	c. 5202_5203delGT	p. F1735Qfs*6		0
	ex44	c. 8439_8442dupTGCA	p. E2815Cfs*19		0
14	ex26	c. 5202_5203delGT	p. F1735Qfs*6		0
	ex44	c. 8439_8442dupTGCA	p. E2815Cfs*19		0
15	ex26	c. 4957_4958insA	p. S1653Kfs*2		0
	ex32	c. 6563T>C	p. I2188T		0
16	ex26	c. 4957_4958insA	p. S1653Kfs*2		0
	ex32	c. 6563T>C	p. I2188T		0
17	ex26	c. 4957_4958insA	p. S1653Kfs*2		0
	ex44	c. 8868C>A	p. Y2956*		0
18	ex4	c. 228G>C	p. Q76H		0
	ex16	c. 2522_2523insA	p. Y841fs*		0
19	ex26	c. 4957_4958insA	p. S1653Kfs*2		0
	ex44	c. 8868C>A	p. Y2956*		0
20	ex26	c. 4957_4958insA	p. S1653Kfs*2		0
	ex44	c. 8868C>A	p. Y2956*		0
21	ex26	c. 4957_4958insA	p. S1653Kfs*2		0
	ex41	c. 8048C>T	p. T2683I		0
22	ex26	c. 4957_4958insA	p. S1653Kfs*2		0
	ex44	c. 8868C>A	p. Y2956*		0
23	IVS27	c. IVS27-2A>G	p. -		0
	ex44	c. 8299G>C	p. D2767H		0
24	ex10	c. 2101C>T	p. P701S		0
	ex26	c.4557delA	p. A1520Pfs*30		0
25	IVS27	c. IVS27-3_4insT	p. -		0
	ex4	c. 35T>C	p. M12T		0
26	ex35	c.6869_6896delccatattcctgcaaatgttcaaattcataagaaag	2290Qfs*12		0
	ex26	c. 4957_4958insA	p. S1653Kfs*2		0
27	ex32	c. 6563T>C	p. I2188T		0
	ex35	c. 6897_6902 dupAGGTCC	p. G2300_P2301dup		0

28	ex32	c. 6557G>A	p. G2186E	rs74419361	0.0023
	ex35	c. 6976C>T	p. R2326Q	rs4710457	0.3496
29	ex4	c. 141A>T	p. E47D		0
	ex26	c. 4957_4958insA	p. S1653Kfs*2		0
30	ex26	c. 4957_4958insA	p. S1653Kfs*2		0
	ex32	c. 6557G>A	p. G2186E	rs74419361	0.0023
31	ex26	c. 4957_4958insA	p. S1653Kfs*2		0
	ex44	c. 8351T>G	P. L2784R		0

Thirty one patients were found to carry compound heterozygous *EYS* mutations.
There were no patients who carry a homozygous *EYS* mutation.
* indicates truncating and nonsense variants.
